# Small bowel enteroclysis with magnetic resonance imaging and computed tomography in patients with failed and uncertain passage of a patency capsule

**DOI:** 10.1186/1471-2342-12-3

**Published:** 2012-02-15

**Authors:** Frans-Thomas Fork, Nils Karlsson, Sattar Kadhem, Bodil Ohlsson

**Affiliations:** 1 Department of Clinical Sciences Malmö, Medical Radiology, Diagnostic Centre of Imaging and Functional Medicine, Skåne University Hospital, SE-20502 Malmö, Sweden; 2 Medical School at Lund University, BMC Studentcentrum, Se-221 84 Lund, Sweden; 3 Department of Clinical Sciences Lund, Medical Radiology, Skåne University Hospital, SE-221 85 Lund, Sweden; 4 Department of Clinical Sciences Malmö, Division of Gastroenterology, Skåne University Hospital, SE-20502 Malmö, Sweden

**Keywords:** Small bowel, enteroclysis, magnetic resonance imaging, computed tomography, patency capsule, video capsule enteroscopy

## Abstract

**Background:**

Video capsule enteroscopy (VCE) has revolutionized small bowel imaging, enabling visual examination of the mucosa of the entire small bowel, while MR enteroclysis (MRE) and CT enteroclysis (CTE) have largely replaced conventional barium enteroclysis. A new indication for MRE and CTE is the clinical suspicion of small bowel strictures, as indicated by delayed or non-delivery of a test capsule given before a VCE examination, to exclude stenosis. The aim of this study was to determine the clinical value of subsequent MRE and CTE in patients in whom a test capsule did not present itself in due time.

**Methods:**

Seventy-five consecutive patients were identified with a delayed or unnoticed delivery of the test capsule. Seventy patients consented to participate and underwent MRE (44) or CTE (26). The medical records and imaging studies were retrospectively reviewed and symptoms, laboratory results and imaging findings recorded.

**Results:**

Lesions compatible with Crohns disease were shown by MRE in 5 patients, by CTE in one and by VCE in four, one of whom had lesions on MRE. In patients without alarm symptoms and findings (weight loss, haematochezia, anaemia, nocturnal diarrheoa, ileus, fistula, abscess and abnormal blood tests) imaging studies did not unveil any such lesion. VCE's were performed in only 20 patients, mainly younger than 50 years of age, although no stenotic lesion was shown by MRE and CTE. In the remaining 50 patients no VCE or other endoscopic intervention was performed indicating that the referring physician was content with the diagnostic information from MRE or CTE.

**Conclusion:**

The diagnostic value of MRE and CTE is sufficient for clinical management of most patients with suspected small bowel disease, and thus VCE may be omitted or at least postponed for later usage.

## Background

The least invasive imaging tool that demonstrates the mucosa of the whole small bowel in detail is the video capsule for enteroscopy (VCE). The capsule is an environmentally friendly, high-tech miniature endoscope, designed to be swallowed for spontaneous passage through the small bowel. VCE soon established itself as a first-line diagnostic procedure of small bowel diseases [1,2] although it carries a small risk of the capsule getting stuck in a stenosis. In order to avoid this calamity, a self-disintegrating sham capsule, known as the patency capsule (PC) and equal in size and shape to the video capsule, was soon introduced to prove a non-obstructed small bowel passage. The capsule is naturally excreted within 18 hours. If not noted, a dedicated screening tool for external abdominal application is used to trace it. If the capsule is found to be present or the test is equivocal, a radiological examination of the small bowel is performed to rule out any stenotic lesion before a diagnostic VCE is contemplated. A retained PC will spontaneously disintegrate after 36 hours and thus prevent bowel obstruction.

Radiology of the small bowel is today performed as an enteroclysis, either by means of magnetic resonance imaging (MRE) or computed tomography (CTE). Both techniques are established in routine practice [3] and are highly sensitive for the detection of small bowel lesions as well as extra-intestinal complications such as abscesses, fistulae and involvement of other organs [4-8]. MRE has the advantage of combining high diagnostic performance and lack of ionizing radiation and this is reflected in increasing clinical demand. We decided to determine the diagnostic outcome of a small group of patients in whom a PC did not present itself in due time. These patients all underwent MRE and CTE. This series is part of our continuous audit of imaging techniques, diagnostic quality and outcome [9].

Our hypothesis was that after an unnoticed passage of the PC per rectum or true impaction of the PC, the diagnostic value of follow-up examination with MRE or CTE is sufficiently comprehensive for the clinician to waive or postpone the intended VCE for later use. To the best of our knowledge, no such information is available in the literature. Thus, the aim of this study was to define the clinical value of subsequent MRE and CTE in patients in whom a test capsule did not present itself in due time.

## Methods

All medical data were dealt with in agreement with the ethical principles for medical research established in the Helsinki Declaration of 1975 and as per standard practice at our department. The study was approved by the Ethics committee of Lund University and patients gave their informed consent to let us review their clinical files and small bowel images for this retrospective review.

### Study design

Consecutive out-patients from the Department of Gastroenterology at our University Hospital with clinically suspected or known disease of the small bowel and who were referred for VCE, were retrospectively included in this study. All had an initial PC test that resulted in a late or unconfirmed excretion of the capsule. These patients were subsequently examined with either MRE or CTE. Patient recruitment extended over a three year period from January 1, 2005, until January 31, 2008, with follow-up until mid 2010. Of seventy five consecutive patients invited by letter seventy agreed to participate.

Images were stored on Sectra PACS and reviewed on a Siemens work station. All appropriate medical records were reviewed and the following data were recorded: gender, age at clinical consultation, duration and type of symptoms (abdominal pain, diarrhoea, weight loss, nausea and anaemia), laboratory analyses (hemoglobin, C-reactive protein (CRP), leukocytes, orosomucoid and albumin in serum, and calprotectin in faeces), indication for the intended VCE, passage delay of PC, and type, quality and report of imaging studies. Duration of symptoms until the time of the PC test, often only given as an estimate in the medical files, was classified as less than 1 year, up to 2.5 years and longer than 2.5 years. The following symptoms and signs were classified as alarm symptoms: weight loss, haematochaezia, anaemia, nocturnal diarrhoea, ileus, fistulae and abscesses. When a VCE had been performed after the PC, its indication was registered and the VCE based diagnosis was considered to be the true one. Time between examinations was calculated.

The diagnostic accuracy of MRE and CTE were calculated, defining the true diagnosis as the one based on endoscopic follow-up procedures, or on clinical observations after the latest imaging procedure for up to 24 months.

### Patient characteristics

Seventy patients with a non-documented passage of the PC, 50 female and 20 male, mean age 45 years, range 10-83 years (Table 1), were included in this series. Five patients had difficulty in swallowing the PC, and one patient with neurogenic swallowing dysfunction had the PC deposited in the duodenum through gastroscopic deliverance. The clinical indications for the intended VCE were suspected Crohn's disease in 47 patients, small bowel bleeding in 13 patients and abdominal discomfort, mainly related to bowel dysfunction, in 10 patients.

**Table 1:** Number of patients related to age, gender and method of enteroclysis

Age group (years)	N	MRI		CT		Final VCE	
		**Male**	**Female**	**Male**	**Female**	**Male**	**Female**

00-29	22	3	19	-	-	2	8

30-49	23	5	12	3	3	4	4

50+	25	2	3	7	13	2	3

***TOTAL***	***70***	***10***	***34***	***10***	***16***	***8***	***15***

N = number of patients, MRI = magnetic resonance imaging, CT = computed tomography, VCE = video capsule endoscopy.

**Figure 1:**
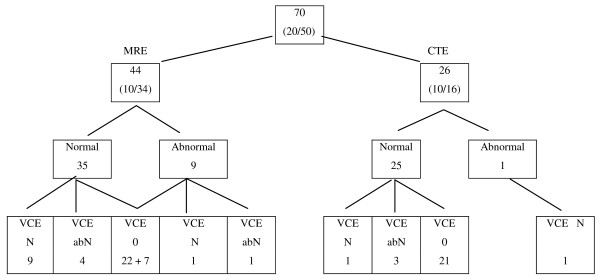
**Flow chart of patients and examinations**. Figures denote number of patients and gender (male/female). N = normal; abN = abnormal; 0 = not examined. A final video capsule enteroscopy (VCE) was not considered necessary after magnetic resonance imaging (MRI)- and computed tomography (CT) enteroclyses in 71% (50/70 patients).

The patient history was less than 12 months in 22 patients, up to 2.5 years in 24 others, and longer in 24 patients. The distribution of patients to small bowel findings of enteroclyses and VCEs is given in Figure 1.

### MR and CT enteroclysis

The following technical considerations were valid for both methods. Patients were allowed free fluid but otherwise nil by mouth after midnight. After lubrication of the nasal mucosa with 2% lidocaine hydrochloride gel (Xylocaine^®^, AstraZeneca, Gothenburg, Sweden), a 13 French Gauge naso-jejunal catheter (insert manufacturer) was advanced under fluoroscopic guidance beyond the duodeno-jejunal junction. No conscious sedation was used. Adequate small bowel luminal distension was achieved by infusing an iso-osmotic polyethylene glycol solution which is neutral on CT images (HU = 0), black on T1-weighted MR images and white on T2-weighted. The infusion rate was adjusted to 100 ml/min to a total of 1500 +/- 250 ml, and administered via an electric roller pump (Watson Marlow 323^®^, Wilmington, MA, USA). Lesion detection was improved by the administration of an intravenous contrast agent. Scanning commenced when caecal filling was established and peristalsis eliminated (20 mg Buscopan^® ^(Boeringer Ingelheim, Stockholm, Sweden) or 0.3 mg Glucagon^® ^(Novo Nordic, Malmö, Sweden) intravenously).

### MRE

MRI was performed on a 1.5 Tesla MR system (Magnetom Symphony, Siemens Medical Solutions, Erlangen, Germany), using a phased array coil with four elements being placed on the abdomen. The infusion of the polyethylene solution was monitored by a heavily T2-weighted, single-shot turbo spin-echo sequence for MR fluoroscopy, and motility was studied with coronal dynamic true fast imaging with steady-state precision (true FISP) sequence [10]. Small bowel morphology was examined with a true FISP sequence (TR/TE 6 ms/3 ms, slice thickness 3 mm) and HASTE images (TR/TE = infinite/90 ms, slice thickness 5 mm) applied in axial and coronal planes (image matrix 256 × 256). Following intravenous application of Gd-DOTA meglumine (Dotarem^®^, Guerbet, Aulnay-sous-Bois, France) in a dose of 0.2 mmol/kg body weight, a coronal 2D gradient-echo (FLASH) sequence with fat saturation (TR/TE = 103/7.2 ms, thickness 4 mm, image matrix 256 × 512) was applied during breath holding [6].

### CTE

All patients received an intravenous injection of 120 ml of iohexol (Omnipaque^®^, GE Healthcare, Oslo, Norway) with an iodine concentration of 300 mgJ/ml. The rate of injection was set at 4 ml/s via an automatic power injector followed by a saline flush of 50 ml. The volumetric acquisition was performed in the late arterial dominant phase, 35 s after the start of injection. CT scanning was performed on a multi channel detector CT (Somatom Sensation 16, Siemens Medical Systems, Erlangen, Germany) in prone position, from dome of diaphragm to pubic symphysis. Scan parameters were 16 × 0.75 mm collimation with a reconstruction interval of 1.0 mm, rotation time 0.5 s, tube voltage 100 kV and 240 mAs. Reformats at 3 mm in transverse, coronal and sagittal planes were sent to the picture archiving system [11-14].

### Evaluation of enteroclysis

All MRE and CTE examinations were re-evaluated by two radiologists in consensus, one of whom was board-certified and specialized in gastrointestinal radiology and endoscopy, and the other in his final training. The overall image quality and small bowel distension was defined as good, sufficient or insufficient separately for the duodenum and jejunum, the proximal and mid-ileum, and the distal and terminal ileum.

The enteroclyses were all re-classified and the following features were looked for: bowel wall thickness and masses, stenotic lesions, pre-stenotic dilatation, regional contrast enhancement of the mucous membrane and gut wall as compared to unaffected parts and nearby vessels, mucosal surface irregularities, mural and transmural involvement, fissures, engorged vessels and fibro-fatty proliferation of the mesentery and abnormalities of the peritoneum. Radiologic signs were summarized into three groups, those compatible with Crohn's disease, an alleged bleeding source, i.e. a tumour or an ulcer and miscellaneous findings such as adhesions, benign adenopathy, impaired peristalsis in an otherwise normal small bowel and other non-specific observations.

### Video capsule enteroscopy (VCE)

The Given Diagnostic Imaging System^® ^(Yoqneam, Israel) consists of the PillCam SB capsule, which includes a miniature video camera, an external antenna with attached portable computer, and a workstation with appropriate software for review, interpretation, and reporting of images. The capsule provides two frames per second over about eight hours, at 1:8 magnification, with a 140^○ ^field and 1 to 30 mm depth of view. The capsule is passively progressed through the gastrointestinal tract and excreted in the natural way. The patients fasted overnight before swallowing the capsule. They were advised to drink liquids after two hours and to have a light meal after four hours. The patients wore a belt with a power supply and a small hard drive for receiving images. Before ingesting the capsule, a sensor array was fixed to the patient's abdominal wall and connected to the hard drive. The patients were encouraged to live as normally as possible during the eight hour video recording. The equipment was returned to the Endoscopy Unit the following day and the data downloaded to a computer for evaluation. The video recordings, featuring more than 50 000 images, were reported on a workstation. After an evaluation requiring about one hour, the physician was able to save images and create reports to be viewed and printed. The RAPID™ application software also provided a localization diagram of the capsule's progress through the small bowel [15]. A PC was used to establish free passage through the small bowel tract before the proper VCE.

VCE offers a magnified view of the small bowel mucosal lining, i.e. tiny capillaries and small clusters of villi are disclosed in coloured detail. Superficial lesions and any bleeding source are readily detected. A normal lumen width is in most instances easy to evaluate, whereas the reason for a narrowed lumen is more difficult to determine, be it thickened mucosa, fistulous opening or an extra-intestinal manifestation, as VCE cannot reveal any pathologic processes beyond the mucosa itself [16].

### Statistical analyses

Fisher's exact test was used to calculate associations between the presence of alarm symptoms, laboratory analyses and findings on examinations. P < 0.05 was considered statistically significant.

## Results

### MRE and CTE

The series includes 44 MRE and 26 CTE examinations. Fluoroscopy of the abdomen before the MRE's showed no trace of a retained PC. The quality of radiological examinations was rated excellent in 64 cases, sufficient for diagnosis in four (three MRE and one CT examinations) and suboptimal in two female patients, due to breathing artefacts in one and due to reflux of polyethylene glycol solution into the stomach in the other, leaving too little behind for optimal distension of the terminal ileum. One female had a second PC prior to a final VCE, the other was re-evaluated clinically with no further imaging study requested.

No abnormality was found at MRE and CTE in 60 out of 70 patients (Figure 1), including all 13 cases of occult bleeding and nine of 10 cases with abdominal discomfort. Signs of Crohn's disease were revealed in six cases out of 47 with the same clinical diagnosis. Miscellaneous findings were revealed in four other cases on MRI examination where the diagnoses were: adhesions in two patients after bowel resection for Crohn's disease, increased number of normal mesenteric lymph nodes in one and increased contrast enhancement of normal-sized, mesenteric lymph nodes in a fourth patient. Both the latter patients demonstrated normal follow-up VCEs. Pathologic findings to gender are evident from table 2.

**Table 2:** Diagnostic results related to gender, age and method of examination

			Gender		Age groups (years)		
	**Diagnosis**	**Sub-total**	**Male** **(n = 20)**	**Female** **(n = 50)**	**< 30** **(n = 22)**	**30 - 49** **(n = 23)**	**50 >** **(n = 25)**

**MRI**	Normal	35	8	27	18	12	5
			
(n = 44)	CD	5	1	4	3	2	-
			
	Misc.	4	1	3	1	3	-

**CT**	Normal	25	9	16	-	5	20
			
(n = 26)	CD	1	1	-	-	1	-

**VCE**	Normal	12	4	8	7	4	1
			
(n = 23)	CD	4	1	3	3	1	-
			
	Misc.	4	2	2	-	1	3
			
	Absentee	3	1	2	1	1	1

CD = Crohn's disease, M = miscellaneous findings (adhesion, stricture, diverticulae, etc.), MRI = magnetic resonance imaging, CT = computed tomography, VCE = video capsule endoscopy.

### Video capsule enteroscopy

Twenty-three patients were finally referred for a VCE examination (Figure 1). The clinical indications were Crohn's disease in 15 patients; occult bleeding in five patients and other indications in three patients. At study end, no VCE had been carried out in three patients as two refused the examination and one was cancelled by the referring clinician. Hence, 20 VCEs were available for analysis in 15 of 44 patients previously investigated with MRI (34%), and five of 26 patients examined with CT (19%). Half of them were done in young patients up to 30 years of age. Pathologic findings at VCE were documented in five females and three males (Table 2).

Out of 15 patients with clinically suspected Crohn's disease, final VCE verified the disease in four. Only one of them had signs of Crohn's disease on MRI, whilst three had normal imaging studies. Two of these (one MRE and another with CTE), had jejunal erosions on VCE, so far not verified during the follow-up period. On the MRI examination 14 days earlier an increased number of normal mesenteric lymph nodes were reported, but no signs of inflammatory bowel disease and no such signs at a follow-up ileocolonoscopy examination four months later. VCE in the third patient, who had undergone a CTE, showed minimal changes in the duodenum and numerous erosions in the terminal ileum. A follow-up ileocolonoscopy performed two months later revealed tiny petechiae. Histology of biopsies from the terminal ileum revealed lymphatic hyperplasia only.

The VCE revealed erosions and mucosal scarring of NSAID type in one patient, subtle inflammation in the terminal ileum and a tiny ulcerated polypoid lesion in another and non-specific mild ileitis with a single small ulcer in a third patient with normal MRE and ileocolonoscopy studies. The last patient had previously undergone surgical removal of a duodenal cancer and presented herself with gastric retention at VCE. In the five patients with clinically suspected occult bleeding, no abnormalities were revealed at VCE.

### At study end

In 50 of 70 patients (71%) the primarily intended VCE examination was never performed. Of the 10 patients initially referred for "abdominal discomfort", the intended VCE was not performed in eight. The same was true for seven of nine patients with an abnormal MRE (Figure 1). Morphologic signs of abnormality based on one of three imaging techniques were disclosed in 17 patients, 12 under the age of 50 years, five men and 12 women. Pathology was demonstrated by two methods in one male patient. The mean time between the PC test and enteroclysis was three months (range 0 - 18 months), between enteroclysis and final VCE examination 1.5 months, (range 0.5 to four months) in 18 patients, and exceeded 23 months in the last two patients. Seven of 10 patients with abnormal findings had their examinations soon after their referral, but this was not statistically significant.

No correlation between alarm symptoms and morphologic findings could be established by any of the three imaging modalities (data not shown). In contrast, elevated calprotectin levels in faeces were associated with an abnormal VCE (p = 0.009), and pathological laboratory analyses were associated with an abnormal MRE (p = 0.050). All 17 patients without alarm symptoms and with normal laboratory analyses had normal examinations.

### Complications and limitations

The only complication was a non-serious esophageal contact bleeding after gastroscopic delivery of a PC to the duodenal cap in one dysphagic patient. One patient refused VCE because of fear of the capsule getting stuck and two enteroclyses were technically suboptimal.

## Discussion

This study showed that none of the 70 patients referred for a VCE, who had an undelivered initial PC, had gastrointestinal obstruction as proved by follow-up MRE or CTE examinations. Only 20 of the 70 went on to have a VCE.

During the three year study period a total of 714 VCEs were performed. Whilst performing a quality audit we came across a number of patients who had not gone on to VCE because the preceeding PC did not present itself in time. As evident from our results, 60 of 70 consecutive patients with undelivered PC had normal enteroclysis studies. What happened to these patients next? What was the clinician's understanding of our cross-sectional imaging results, and what impact had these on patient management? As no such information was found in the literature, we undertook this retrospective study to get a qualified estimate of the clinical value of our imaging performance.

VCE has proven itself to be the most effective examination in depicting small bowel mucosal lesions, including subtle signs of chronic inflammatory diseases [17] and obscure bleeding [18]. New capsule enteroscopes designed for oesophageal and colonic exploration are being introduced to the market [19]. In patients with a risk of capsule retention (4%), total enteroscopy is still possible in more than four out of five patients [20,21]. Small bowel neoplasia remains rare but half of them might be revealed by VCE, i.e. VCE is diagnostically more efficient than any other imaging modality [22]. The overall diagnostic success with VCE has allowed gastoenterologists to proclaim VCE to be a first line procedure in ruling out small bowel disease [23]. However, the detailed depiction of the inner gut surface with VCE sometimes gives rise to interpretation difficulties as the specificity for detecting Crohn's disease is only 53% [24]. Accordingly, erosions of the small bowel do not ultimately lead to a diagnosis of Crohn's disease [25]. In the present study we encountered three such cases with alleged erosions in the terminal ileum, not confirmed at subsequent ileocolonoscopies.

We were able to make two interesting observations. One was that no organic lesion was diagnosed by us in 60 cases by MRE and CTE, i.e. the imaging results did not mirror the clinical suspicion of active small bowel disease. The other was that in most cases the gastroenterologist did not proceed with the primarily intended VCE examination, neither to confirm imaging results nor to verify their preliminary clinical suspicion. It was evident from the medical notes that results of MRE or CTE, together with a clinical re-evaluation of the patient's situation, gave the gastroenterologist sufficient data not to undertake further enteroscopies.

The reason for the low number of patients diagnosed with Crohn's disease with either CTE or MRE in our series is not known, but has probably to do with image resolution. Erosions, typical for Crohn's disease, are superficial and shallow; viz. lesions limited to the mucosa are not possible to unveil by these methods. However, the clinical value of modern enteroclysis examinations seems to be sufficient as few of the primarily intended VCE examinations were done. The true clinical value is still pending, but our follow-up of up to 24 months has yet not revealed any new disease, indicating the absence of false negative imaging studies.

In a patient with a biopsy-proven diagnosis of inflammatory bowel disease, future examinations of the bowel are more focused on complications than on confirmation of diagnosis. In a case like that, cross-sectional imaging with MRE or CTE ought to be performed before deciding on a VCE, saving the costs for at least a PC. Furthermore, a strategy that saves a VCE examination, especially when preceded by a PC, would save health care costs and minimize inconvenience.

In our department, patients under the age of 45 years are allocated to MRE, and older ones to CTE. As Crohn's disease mostly starts in a younger patient group, signs of active Crohn's disease are found more often with MRI than with CT. A diagnosis of Crohn's disease is established when verified endoscopically and proven by histology [26]. Early diagnosis of is important as tailored treatment may reverse active disease and prevent complications. Younger patients with symptoms attributed to the small bowel are likely to harbour early and superficial lesions, that might be difficult to reveal on MRE and CTE examinations, but visible on VCE. A thorough diagnosis is difficult in early disease, mirrored in our series by the observation that referring doctors seemed to be more prone to send younger patients to a final VCE after MRE (15 cases) than after CTE (five cases). Elective imaging of the small bowel is done as an enteroclysis procedure in our institution, i.e. with a naso-jejunal tube, because this allows the radiologist to achieve optimal bowel filling for maximal diagnostic performance [10]. Our examination techniques are on a par with those of leading European experts [6] which means that our results apply to MRI and CT enteroclyses and not to MRI or CT enterographies in which patients are given contrast by mouth.

All patients were selected and referred by gastroenterologists from one hospital. The fact that the sample population consisted of 2.5 times as many females (50/20) suggests that there might be an imbalance in the inclusion process of patients with a failed PC test. The skewed selection may partly be explained by the limited size of our sample population. Furthermore, it has been shown that women have longer bowel transit times than men, which may indicate that the acceptable PC transit time in women ought to be longer than the 48 h stated [27].

Given the limited size of our material sample, e.g. only eight cases of established Crohn's disease, solid statistical analyses of the temporal aspects of various diagnostic delays were not possible. However, patients diagnosed with Crohn's disease had examinations performed closer in time than the others. As might be expected, normality of the imaging studies in patients with mild to moderate clinical symptoms, i.e. absence of alarm symptoms, may also be explained by the fact that many of these patients were admitted to examination although their laboratory analyses were close to normal. A limitation of this retrospective study is that not all patients had a VCE. As only 29% of our patients had a final VCE we had to accept the clinical follow-up diagnoses as our "gold standard". One might comment that the absence of a final VCE proved our case, viz. gastroenterologists did not anticipate any further diagnostic information of importance for managing 71% of their patients besides the information obtained by the enteroclysis examinations.

## Conclusion

Based on the observation in our defined group of patients it seems justified to conclude that the diagnostic value of MRE and CTE studies is sufficient for the clinical management of patients with alleged small bowel disease. As a consequence, MRE and CTE enteroclyses might be chosen as first line examination methods of the small bowel, followed by VCE's when clinically indicated. This might save health care resources for the better use in other patients. Patients without alarm symptoms and pathological laboratory analyses should primarily not be referred to further examinations.

## Lists of Abbreviations

CT: Computed tomography; MRI: Magnetic resonance imaging; CTE: Computed tomography enteroclysis; MRE: Magnetic resonance enteroclysis; PC: Patency capsule; VCE: Video capsule enteroscopy; SB: Small bowel; CRP: C-reactive protein; FISP: Fast imaging with steady state precision.

## Competing interests

The authors declare that they have no competing interests.

## Authors' contributions

FTF: Initiating the study, tutoring NK and SK, finalizing the manuscript. NK: Collecting clinical and imaging data. SK: Second reader of imaging studies. BO: Interpreting medical data, statistical analysis, contributing to the manuscript. All authors have read and approved the final manuscript.

## Pre-publication history

The pre-publication history for this paper can be accessed here:

http://www.biomedcentral.com/1471-2342/12/3/prepub

## References

[B1] Hara AK Leighton JA Sharma VK Fleischer DE Small bowel: preliminary comparison of capsule endoscopy with barium study and CT Radiology 2004 230 260 265 10.1148/radiol.2301021535 14617764

[B2] Fork FT Aabakken L Capsule entereoscopy and radiology of the small intestine Eur Radiol 2007 17 3103 3111 10.1007/s00330-007-0718-7 17876583

[B3] Schmidt S Lepori D Meuwly JY Duvoisin B Meuli R Michetti P Felley C Schnyder P van Melle G Denys A Prospective comparison of MR enteroclysis with multidetector spiral-CT enteroclysis: interobserver agreement and sensitivity by means of "sign-by-sign" correlation Eur Radiol 2003 13 1303 1311 12764646 10.1007/s00330-002-1710-x

[B4] Umschaden HW Szolar D Grasser J Umschaden M Haselbach H Small-bowel disease: comparison of MR enteroclysis images with conventional enteroclysis and surgical findings Radiology 2000 215 717 725 10831690 10.1148/radiology.215.3.r00jn12717

[B5] Wiarda BM Kuipers EJ Heitbrink MA van OA Stoker J MR enteroclysis of inflammatory small-bowel diseases Am J Roentgenol 2006 187 522 531 10.2214/AJR.05.0511 16861559

[B6] Gourtsoyiannis N Papanikolaou N Grammatikakis J Papamastorakis G Prassopoulos P Roussomoustakaki M Assessment of Crohn's disease activity in the small bowel with MR and conventional enteroclysis: preliminary results Eur Radiol 2004 14 1017 1024 10.1007/s00330-004-2302-8 15057562

[B7] Lasocki A Pitman A Williams R Lui B Kalade AV Farish S Relative efficacy of different MRI signs in diagnosing active Crohn's disease, compared against a histological gold standard J Med Imaging Radiat Oncol 2011 55 11 19 10.1111/j.1754-9485.2010.02223.x 21382184

[B8] van Weyenberg SJB Meijerink MR Jacobs MA van der Peet DL van Kuijk C Mulder CJ van Waesberghe JH MR enteroclysis in the diagnosis of small-bowel neoplasms Radiology 2010 254 765 773 10.1148/radiol.09090828 20177091

[B9] Negaard A Mulahasanovic A Reisaeter A Aasekjaer K Sandvik L Klow N-E Crohn's disease evaluated with magnetic resonance enteroclysis: diagnostic performance of experienced and inexperienced readers before and after training Acta Radiol 2008 49 967 974 10.1080/02841850802409539 18925449

[B10] Gourtsoyiannis N Papanikolaou N Grammatikakis J Prassopoulos P MR Enteroclysis: technical considerations and clinical applications Eur Radiol 2002 12 2651 2658 12386753 10.1007/s00330-002-1507-y

[B11] Schmidt S Lepori D Meuwly JY Duvoisin B Meuli R Michetti P Felley C Schnyder P van Melle G Denys A Prospective comparison of MR-enteroclysis (MRE) with multidetector spiral-CT-enteroclysis (MSCTE) Eur Radiol 2002 13 1303 1311 12764646 10.1007/s00330-002-1710-x

[B12] Maglinte DD Bender GN Heitkamp DE Lappas JC Kelvin FM Multidetector-row helical CT enteroclysis Radiol Clin North Am 2003 41 249 262 10.1016/S0033-8389(02)00115-X 12659337

[B13] Patak MA Mortele KJ Ros PR Multidetector row CT of small bowel Radiol Clin North Am 2005 43 1063 1077 10.1016/j.rcl.2005.07.009 16253662

[B14] Paulsen SR Huprich JE Fletcher JG Booya F Young BM Fidler JL Johnson CD Barlow JM Earnest F CT enterography as a diagnostic tool in evaluating small bowel disorders: review of clinical experience with over 700 cases Radiographics 2006 26 641 657 discussion 657-662 10.1148/rg.263055162 16702444

[B15] Iddan G Meron G Glukhovsky A Swain P Wireless capsule endoscopy Nature 2000 405 417 10839527 10.1038/35013140

[B16] Halligan S Saunders B William C Bartram C Adult Crohn's disease: can ileoscopy replace small bowel radiology? Abdom Imaging 1998 232 117 121 10.1007/s002619900301 9516495

[B17] Albert JG Martiny F Krummenerl A Stock K Lesske J Gobel CM Lotterer E Nietsch HH Behrmann C Fleig WE Diagnosis of small bowel Crohn's disease: a prospective comparison of capsule endoscopy with magnetic resonance imaging and fluoroscopic enteroclysis Gut 2005 54 1721 1727 10.1136/gut.2005.069427 16020490 PMC1774782

[B18] Saurin JC Delvaux M Gaudin JL Fassler I Villarejo J Vahedi K Bitoun JM Souquet JC Ponchon T Florent C Gay G Diagnostic value of endoscopic capsule in patients with obscure digestive bleeding: Blinded comparison with video push-enteroscopy Endoscopy 2003 35 576 584 12822092 10.1055/s-2003-40244

[B19] Eliakim R Fireman Z Gralnek IM Yassin K Waterman M Kopelman Y Lachter J Koslowsky B Adler SN Evaluation of the PillCam Colon Capsule in the detection of colonic pathology: results of the first multicenter, prospective, compararative study Endoscopy 2006 38 963 970 10.1055/s-2006-944832 17058158

[B20] Toth E Fork FT Almqvist P Thorlacius H Endoscopy-assisted capsule endoscopy in patients with swallowing disorders Endoscopy 2004 36 746 747 reply 747-748 10.1055/s-2004-825685 15280990

[B21] Lopes S Figueiredo P Portela F Freire P Almeida N Lérias C Gouveia H Leitão MC Capsule endoscopy in inflammatory bowel disease type unclassified and indeterminate colitis serologically negative Inflamm Bowel Dis 2010 16 1663 1668 10.1002/ibd.21249 20848457

[B22] Cheung DY Lee IS Chang DK Kim JO Cheon JH Jang BI Kim YS Park CH Lee KJ Shim KN Ryu JK Do JH Moon JS Ye BD Kim KJ Lim YJ Choi MG Chun HJ Korean Gut Images Study Group Capsule endoscopy in small bowel tumors: a multicenter Korean study J Gastroenterol Hepatol 2010 25 1079 1086 10.1111/j.1440-1746.2010.06292.x 20594222

[B23] May A Manner H Schneider M Ipsen A Ell C Prospective multicenter trial of capsular enodsocopy in patients with chronic abdominal pain, diarrhea and other signs and symptoms (CEDAP_Plus study) Endocopy 2007 39 606 612 10.1055/s-2007-966640 17611915

[B24] Solem CA Loftus EV Jr Fletcher JG Baron TH Gostout CJ Petersen BT Tremaine WJ Egan LJ Faubion WA Schroeder KW Pardi DS Hanson KA Jewell DA Barlow JM Fidler JL Huprich JE Johnson CD Harmsen WS Zinsmeister AR Sandborn WJ Small-bowel imaging in Crohn's disease: a prospective, blinded, 4-way comparison trial Gastrointest Endosc 2008 68 255 266 10.1016/j.gie.2008.02.017 18513722

[B25] Ohlsson B Bengtsson M Nielsen J Toth E A prospective evaluation of the diagnostic value of video capsule endoscopy in patients initially classified as irritable bowel syndrome Eur J Int Med 2009 20 48 52 10.1016/j.ejim.2008.04.018 19237092

[B26] Nikolaus S Schreiber S Diagnostics of inflammatory bowel disease Gastroenterology 2007 133 1670 1689 10.1053/j.gastro.2007.09.001 17983810

[B27] Sadik R Abrahamsson H Stotzer P-O Gender differences in gut transit shown with a newly developed radiological procedure Scand J Gastroenterol 2003 38 36 42 10.1080/00365520310000410 12608462

